# Wound Irrigation in the Prevention of Surgical Site Infection in Elective Colorectal Surgery: A Retrospective Cohort Study

**DOI:** 10.7759/cureus.64662

**Published:** 2024-07-16

**Authors:** Sahil S Shet, Helen Earley, Ben Creavin, Aryan S Shet, Cliodhna NicGabhann, Peter McCullough, Fiachra Cooke, Peter Neary

**Affiliations:** 1 Department of General and Colorectal Surgery, University Hospital Waterford, Waterford, IRL; 2 GKT School of Medical Education, King’s College London, London, GBR

**Keywords:** hemicolectomy, surgical site infection, wound irrigation, pulse lavage, colorectal surgery

## Abstract

Background

Surgical site infection in colon surgery is associated with significant cost and increased length of hospital stay. Recently, there has been interest in the use of pulsed lavage to reduce the risk of surgical site infection in contaminated wounds. Although increasingly used and gaining popularity, its effectiveness in elective colorectal surgery has been poorly documented. This study aimed to investigate the incidence of surgical site infection within 30 days of elective colorectal surgery in patients who underwent wound irrigation with pulse lavage versus standard closure.

Methodology

A retrospective study was conducted at a university hospital over a two-year period between January 2020 and December 2021. All adult patients who underwent elective colorectal surgery were eligible for inclusion.

Results

A total of 222 patients underwent elective colorectal surgery during the study period. Operative procedures included abdominoperineal resections, left and right hemicolectomies, pelvic exenterations, small bowel or large bowel resections, as well as stoma reversals, formations, and refashioning. In total, 76 patients underwent pulse lavage while 146 did not. The total number of surgical site infections was 39 during the study period. Infection rates in the pulse lavage group were 14.47% compared to 19.18% in the standard closure group. The chi-square analysis concluded the difference in infection rates was not statistically significant (p = 0.213).

Conclusions

The findings demonstrated a difference in infection rates of almost 5% favouring the pulse lavage group; however, it did not reach a statistical difference. Although infection rates were in keeping with those described in the literature, further studies in the form of randomized controlled trials should be performed to determine the benefits, if any, of pulse lavage in colorectal surgery.

## Introduction

Colorectal surgery has one of the highest rates of postoperative surgical site infections (SSIs) among all surgical fields with rates as high as 30% [1,2]. The European Centre for Disease Prevention and Control defines SSIs as postoperative infections occurring within 30 days of a surgical procedure. SSIs place a substantial financial burden on the healthcare system and lead to increased patient morbidity and mortality as well as reduced patient quality of life. This financial burden is a result of increased hospital stays, re-admissions, diagnostic tests, and treatments [3].

Evidence-based skin care bundles are in use across all surgical specialties to reduce the risk of SSIs. Elements of these bundles, including the use of antibiotic prophylaxis, antiseptic skin preparation, and maintenance of normothermia via bear huggers/warming blankets [4], have widespread acceptance; however, the choice of suture material and method of skin closure remains controversial [5,6]. Although the exact costs associated with SSIs in colorectal surgery in Ireland have not been quoted in the literature, globally, the costs of SSIs remain extremely high, with values as high as $3.2 billion in attributable costs yearly being quoted for the United States [7]. Indeed, a 2017 systematic review on the impact of SSIs on healthcare costs across six major European countries found that SSIs are extremely costly and recommended rigorous procedures to be implemented to minimise SSIs.

Studies have shown that pulse lavage irrigation of surgical sites, especially gastrointestinal operations, can significantly reduce the risk of SSI. Although in emergency colorectal surgery the results around SSIs are more clear, the data on elective colorectal surgery is sparse [8-10].

Therefore, this study was conducted to determine whether one of two commonly used wound closure methods, which utilised pulse lavage, offered any advantage over the other in minimising SSI risk, reducing healthcare costs, and improving patient outcomes.

## Materials and methods

Patient identification

This is a single-site retrospective cohort study. All patients undergoing elective bowel resections and stoma operations between 2020 and 2021 at a university hospital in the southwest region of Ireland were included. Operations were performed by one of three consultant colorectal surgeons. Both open and laparoscopic cases were included.

**Figure 1:**
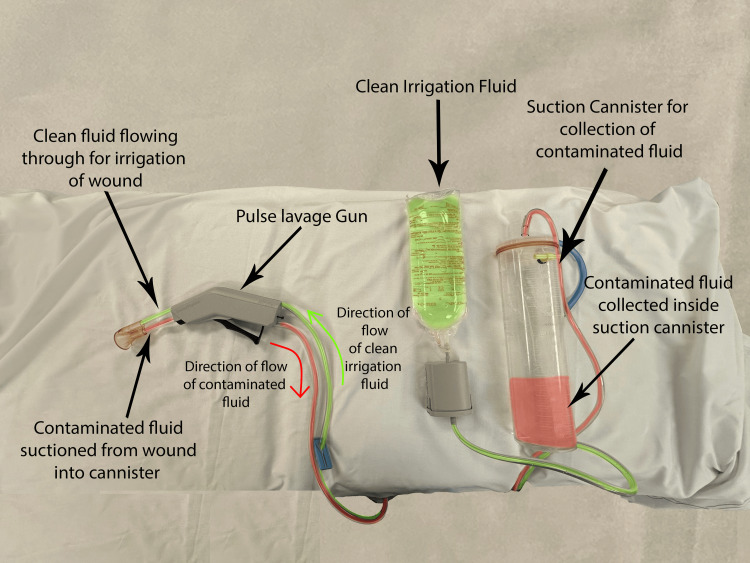
Pulse lavage irrigation with suction. Authors’ own image.

Patients were identified via theatre lists that were compiled by the respective consultants and by cross-referencing these lists with the operation theatre records book. All eligible patients were then divided into two groups depending on the method of wound closure. Group 1 included two of the three consultants, both of whom utilised PDS to fascia and skin clips to the skin (the standard closure group), while Group 2 included the third consultant who utilised pulse lavage with 3 L of 0.9% NaCl and closure PDS to fascia and 2/0 Vicryl® (polyglactin 910) and 3/0 Monocryl® (poliglecaprone 25) to subcutaneous tissue and skin, respectively (the pulse lavage group). Pulse lavage utilised standard equipment and was used on the low-pressure irrigation setting before the closure of the fascia. The pulse lavage irrigation technique is illustrated in Figure 1.

Across the two years, a total of 146 patients were identified in the standard closure group and 76 were identified in the pulse lavage group.

Preoperative surgical preparation

Both groups of patients were prepared in the same manner preoperatively as standardised protocols exist in the hospital and are followed by all three surgeons. The standardised protocol included skin sterilisation, bowel preparation, and preoperative antibiotics. The skin preparation used was a 2% chlorhexidine isopropanol solution for all patients. Bowel preparation was in the form of two sachets of Plenvu taken the night before surgery along with neomycin 1 g and 400 mg metronidazole. Antibiotics were also administered parenterally for all patients and hospital guidelines were 1.2 g IV co-amoxiclav in most patients; however, a combination of IV gentamicin and IV metronidazole was used in patients with a penicillin allergy. For all patients, parenteral antibiotics were administered 30 minutes before skin incision but were not continued routinely postoperatively. Bowel preparation was used for all left-sided colectomies; however, it was not used in stoma reversal surgeries or right-sided colectomies. The exception to this was a reversal of Hartmann’s procedure where standard bowel preparation was used. Iodine-impregnated wound protectors were used for both open and laparoscopic surgeries.

Infection identification

For the purposes of this study, the “infection” group included all patients from the standard closure and pulse lavage groups who met the following criteria within 30 days of the index operation: (1) Confirmed SSI on a wound swab OR (2) required antibiotics for their SSI OR (3) required negative-pressure wound therapy via a V.A.C® device for wound dehiscence. Collections and abscesses were excluded.

The identification of the patients in the “infection” group was done via cross-referencing three main sources. First, the data from all morbidity and mortality (M&M) meetings from 2020 and 2021 were analysed and any identified patients who fit the criteria for the infection group were included. Second, the cloud-based clinical documentation software T-Pro (used by all three colorectal surgery consultants in this study to transcribe and document outpatient clinic reviews) was used to identify patient infections which may not have been captured during the M&M meetings. Lastly, all remaining patients, not already identified as being in the infection group by either M&M meetings or T-Pro, were searched on the iLab Web Enquiry system, which contains information on blood, pathology, and microbiology results. Any patient with a positive wound culture swab on iLab, taken from the surgical site and within 30 days of the index operation, was included in the infection group. The Pearson chi-square test was used to calculate the significance of the difference in infection rates between Group 1 and Group 2. The null hypothesis was that there was no significant difference in the incidence of SSI between the pulse lavage and standard closure groups. A p-value <0.05 was considered significant.

## Results

Standard closure included 72 patients in 2020 and 74 patients in 2021, totalling 146 patients across the study period. Pulse lavage included 42 patients in 2020 and 34 patients in 2021, totalling 76 patients across the study periods (Table 1).

**Table 1:** Number of patients by type of closure. The data have been represented as n where n is the number of patients.

Year	Standard closure	Pulse lavage	Total
2020	72	42	114
2021	74	34	108
Total	146	76	222

The number of infections by type of closure is shown in Table 2.

**Table 2:** Number of cases of infection by type of closure. The data have been represented as n where N is the number of patients and % is the percentage of patients with infection in that group.

Year	Standard closure infections, N (%)	Pulse lavage infections, N (%)	Total, N (%)
2020	10 (13.89%)	8 (19.05%)	18 (15.79%)
2021	18 (24.32%)	3 (8.82%)	21 (19.44%)
Total	28 (19.17%)	11 (14.47%)	39 (17.57%)

The number of infections by type of operation is shown in Table 3.

**Table 3:** Number of cases of infection by type of operation. The data have been represented as n where n is the number of patients.

Year	Laparoscopic operation infections	Open operation infections	Total
2020	13	5	18
2021	5	16	21
Total	18	21	39

Details of the specific procedures in which infection occurred are shown for the standard closure and pulse lavage groups (Tables 4, 5).

**Table 4:** Surgical procedures leading to infection in the standard closure group in 2020 and 2021. The data are represented as N where N is the number of patients and % is the percentage of total patients with infections in the standard closure group.

Surgical procedures	Number of procedures, N (%)
Laparoscopic surgery	10 (35.7%)
Abdominoperineal resection	2 (7.1%)
Left hemicolectomy	1 (3.6%)
Right hemicolectomy	4 (14.2%)
Small bowel tumor resection	1 (3.6%)
Pelvic exenteration	1 (3.6%)
Panproctocolectomy	1 (3.6%)
Open surgery	18 (64.3%)
Ileal and sigmoid resection	1 (3.6%)
Low anterior resection	1 (3.6%)
Reversal of Hartmann’s procedure	2 (7.1%)
Reversal of ileostomy	1 (3.6%)
Right hemicolectomy	2 (7.1%)
Anterior resection	3 (10.7%)
Stoma reversal	3 (10.7%)
Abdominoperineal resection	2 (7.1%)
Ileocolic resection and anastomosis	1 (3.6%)
Panproctocolectomy	1 (3.6%)
Adhesiolysis and ileocolic anastomosis	1 (3.6%)

**Table 5:** Surgical procedures leading to infection in the pulse lavage group in 2020 and 2021. The data are represented as N where N is the number of patients and % is the percentage of total patients with infections in the pulse lavage group.

Surgical procedures	Number of procedures, N (%)
Laparoscopic surgery	8 (72.7%)
Abdominoperineal resection	4 (36.4%)
Right hemicolectomy	1 (9.1%)
Loop colostomy	1 (9.1%)
Anterior resection	1 (9.1%)
Extended anterior resection and en bloc small bowel resection	1 (9.1%)
Open surgery	3 (27.3%)
Refashioning of an end colostomy	1 (9.1%)
Pelvic exenteration	1 (9.1%)
Abdominoperineal resection	1 (9.1%)

The overall infection rate across the study period in the standard closure group was 19.18% (28/146) while the infection rate in the pulse lavage group was 14.47% (11/76). There was no statistically significant difference in SSIs between the two groups using the Pearson chi-square test (χ^2^ =1.5517, p = -0.21288).

## Discussion

Our findings suggest that, overall, patients in the pulse lavage group had a lower risk of postoperative SSI; however, unfortunately, due to the small number of infections, this did not reach statistical significance.

Of note, patients in the standard closure group had a lower risk of infection compared to the pulse lavage group in 2020; however, the opposite was the case in 2021. This discrepancy could be explained by patient-specific risk factors such as diabetes mellitus, smoking, and higher American Society of Anesthesiologists grades which were not accounted for in this study and could affect a patient’s risk of postoperative SSI.

Interestingly, the risk of SSI increased with laparoscopic operations in the pulse lavage group which is surprising considering this less invasive technique has been shown throughout the literature to have lower risks of infections compared to open operations. However, this finding may be explained by too much fluid in the abdomen that is not fully drained and can lead to abscess formation and increased risk of infection. Some studies have suggested that using gauze instead of conventional irrigation after laparoscopic appendicectomies leads to a lower risk of infection [11].

Although preliminary findings suggest that the use of pulse lavage in open surgeries may reduce the risk of infection, it appears to be associated with a potentially increased risk of infection in laparoscopic surgeries. However, due to the small sample size of the study, these results should be interpreted with caution.

Nevertheless, exploration of techniques to reduce SSI risk is crucial as they are among the most common complications after elective colorectal surgery and place a significant burden on healthcare systems worldwide. Not only do they reduce patient quality of life and increase length of stay in hospital but also cause significant financial burden to both the patient and the healthcare system.

Although pulse lavage devices cost significantly more money than lower-pressure irrigation with a syringe and saline, if the rates are infection are indeed lowered, the overall cost to the healthcare system would be significantly lowered. In the United Kingdom, for example, pulse lavage devices range from £21.03 to £50.54 per unit compared to £0.49 for a 1 L sterile jug and £0.35 for a 50 mL syringe [12]. However, the cost of pulse lavage devices pales in comparison to the cost of a single extra day in the hospital in Ireland which costs approximately €878.

The risk of developing an SSI is strongly linked to the level of bacterial contamination present, and most SSIs are caused by the patient’s endogenous flora [13].

As pulse lavage works by delivering an irrigating solution under pressure and concurrently applying suction, it is thought to aid wound cleansing and remove infectious agents and debris from a wound’s surface [14]. In theory, this should reduce the rate of SSI in colorectal surgery.

However, some concerns have been raised about the potential damage to tissue and the penetration of bacteria deeper into the wound from high-pressure irrigation [15].

In contaminated orthopaedic open fractures, the current evidence suggests no difference in re-operation or infection rates between low-pressure irrigation and pulse lavage. Indeed, low-pressure irrigation is recommended due to its lower cost [16].

Recently, a paper was published in the Journal of the American College of Surgeons in which a panel of 15 expert colorectal surgeons, using a modified Delphi process, developed a consensus on the surgical aspects of SSI prevention. The panel supported the use of wound irrigation with povidone-iodine and not with 0.9% saline, as was used by the pulse lavage group in this study. It also did not specify whether pulsatile lavage was the preferred method of wound irrigation. Moreover, the panel concluded that there was not enough evidence to support the use of staples over sutures or vice versa [17].

The evidence for the use of pulse lavage in SSI prevention in colorectal surgery is lacking, largely due to poor-quality data. However, due to the theoretical benefits, it should be explored further.

The purpose of this study was to investigate whether the two different methods of wound closure used in our hospital, which are also commonly used worldwide, had any difference in SSI rates. While there was an almost 5% difference in SSI rates, favouring the pulse lavage group, unfortunately, the study did not reach statistical significance. Thus, a recommendation to use one method over the other cannot be made using this study.

Limitations

Although every effort was made to capture all SSIs in elective colorectal operations across the study period, the method of collecting this data was not infallible. Indeed, some patients may have been missed due to lost notes, poor-quality T-Pro dictations in outpatient clinics, or poor-quality morbidity and mortality data-keeping. This missing data, if any, has the potential to alter the final results achieved in this study.

As this was a retrospective study based on theatre lists and theatre record books to capture cases, the information on patients’ comorbidities was limited. This compounding factor can significantly alter the conclusion as it is well-studied that factors such as poorly controlled diabetes, increased body mass index, and smoking can increase the risk of SSIs [18].

## Conclusions

SSIs contribute to significantly increased healthcare costs, increased patient morbidity and mortality, and reduced patient quality of life. Although many studies have been published in recent years, the quality of data on the best method of wound closure remains poor. Although this study did not reach statistical significance, there was a clear difference between the two methods of closure. A large-scale, multi-centre randomised control trial would provide much higher quality data and would be recommended to reach a more definite conclusion. Such a study design would also negate many of the limitations associated with this retrospective cohort study.
